# Secondary Dyslipidemia In Obese Children - Is There Evidence For Pharmacological Treatment?

**DOI:** 10.5935/abc.20180155

**Published:** 2018-09

**Authors:** Graciane Radaelli, Grasiele Sausen, Claudia Ciceri Cesa, Vera Lucia Portal, Lucia Campos Pellanda

**Affiliations:** Instituto de Cardiologia / Fundação Universitária de Cardiologia - IC/FUC, Porto Alegre, RS - Brazil

**Keywords:** Dyslipidemias, Child, Obesity, Adolescents, Hydroxymethylglutaryl-CoA Reductase Inhibitors, Cholesterol

## Abstract

**Background:**

Long-term safety, effectiveness and criteria for treatment with statins in children are still unclear in clinical practice. There is very limited evidence for the use of medication to treat children with dyslipidemia secondary to obesity who do not respond well to lifestyle modification.

**Objective:**

Systematic review of randomized clinical trials of statin use to treat children and adolescents with dyslipidemia secondary to obesity.

**Methods:**

We performed a search in PubMed, EMBASE, Bireme, Web of Science, Cochrane Library, SciELO, and LILACS for data to evaluate the effect of statins on: improvement of surrogate markers of coronary artery disease in clinical outcomes of adulthood; increased serum levels of total cholesterol (TC), low-density lipoprotein cholesterol (LDL-C) and apolipropotein B (APOB); and decreased serum levels of high-density lipoprotein cholesterol (HDL-C) from inception to February 2016. Participants were children and adolescents.

**Results:**

Of the 16793 potentially relevant citations recovered from the electronic databases, no randomized clinical trials fulfilled the inclusion criteria for children with dyslipidemia secondary to obesity.

**Conclusions:**

We found no specific evidence to consider statins in the treatment of hypercholesterolemia secondary to obesity in children. The usual practice of extrapolating findings from studies in genetic dyslipidemia ignores the differences in long-term cardiovascular risks and the long-term drug treatment risks, when compared to recommendation of lifestyle changes. Randomized clinical trials are needed to understand drug treatment in dyslipidemia secondary to obesity.

## Introduction

According to the National Survey on Health and Nutrition Examination, 11.7% of adults aged 20-39 years and 41.2% of adults aged 40-64 years had elevated low-density lipoprotein cholesterol (LDL-C) levels.^1^ Recent data shows that the estimated number of adults who have total cholesterol (TC) levels ≥ 240 mg/dL reaches 30.9 million and 32.6% of the adults have hypertension.^2^ In 2011-2012, of 5 boys and girls, 1 had abnormal concentration of TC, high-density lipoprotein cholesterol (HDL-C) or non-high density lipoprotein cholesterol (non-HDL-C). The prevalence of high TC, HDL-C, non-HDL-C is 7.8%, 12.8% and 8.4%, respectively, and 20.2% had abnormal concentration of at least 1 of the 3 measurements.^3^ Dyslipidemia causes have changed in epidemiological studies; previously, genetic disturbances were the most common conditions causing dyslipidemia in children. In the last few decades, dyslipidemia secondary to obesity (DSO) has been increasing.^4^

Drug therapy for high-risk lipid abnormalities resulted in great advances in the prevention and treatment of atherosclerotic diseases in adults.^5^ However, the use of pharmacological therapy in children with secondary dyslipidemia is a subject of controversy. Safety, effectiveness and criteria for statin treatment in children are unclear in clinical practice.^6^ There is limited evidence for medication use in children with DSO that do not respond well to lifestyle modification. The majority of studies with statins refer to children and adolescents with genetic dyslipidemia and higher levels of LDL-C.

The objective of this study is to discuss critically the evidence about the effectiveness, safety and effects of the use of statins in children and adolescents with DSO, based on a systematic review of the literature.

## Methods

This systematic review was performed in accordance with the PRISMA Statement and registered at the PROSPERO under identification CRD42015020530.

The search was conducted in MEDLINE (via PubMed), EMBASE, Bireme, Web of Science, Cochrane Library, SciELO, and LILACS. The search strategy included the terms: "Child", "Adolescent", "Hypercholesterolemia", "Dyslipidemias", "Cholesterol", "Hydroxymethylglutaryl-Coa reductase inhibitors", "Statin", “HDL-C” and “Triglycerides”. Two reviewers (G.R. and G.S.) performed the literature search and study selection independently. Disagreements were solved by consensus or by a third reviewer (L.C.P.).

### Selection criteria

**Types of study:** Randomized clinical trials describing statin therapy for children and adolescents. Participants: children and adolescents (up to 18 years old). Interventions: statins for at least 8 weeks. Target condition: DSO. Outcomes: reduction in the risk factors, TC, LDL-C, apolipropotein B (APOB) and HDL-C, improvement in the coronary artery disease indirect markers and/or clinical outcomes in adult life.

**Search limits:** Language: no language restriction. Time period: from inception to February 2016. Design: Randomized clinical trials. Main outcome: risk factors reduction in the infancy, improvement of coronary artery disease indirect markers in clinical outcomes of adult life. Secondary outcomes: statin effects - elevated plasmatic levels of TC, LDL-C and APOB, decrease in the HDL-C levels.

**Inclusion criteria:** Randomized clinical trial reporting children with statin treatment for at least 8 weeks.

**Exclusion criteria:** Non-blinded treatment duplicated data or absent reporting of considered outcomes.

**Data extraction and quality evaluation:** The CONSORT analysis instrument was used to evaluate methodological quality of the included studies performed by two independent reviewers.

## Results

**Figure 1:**
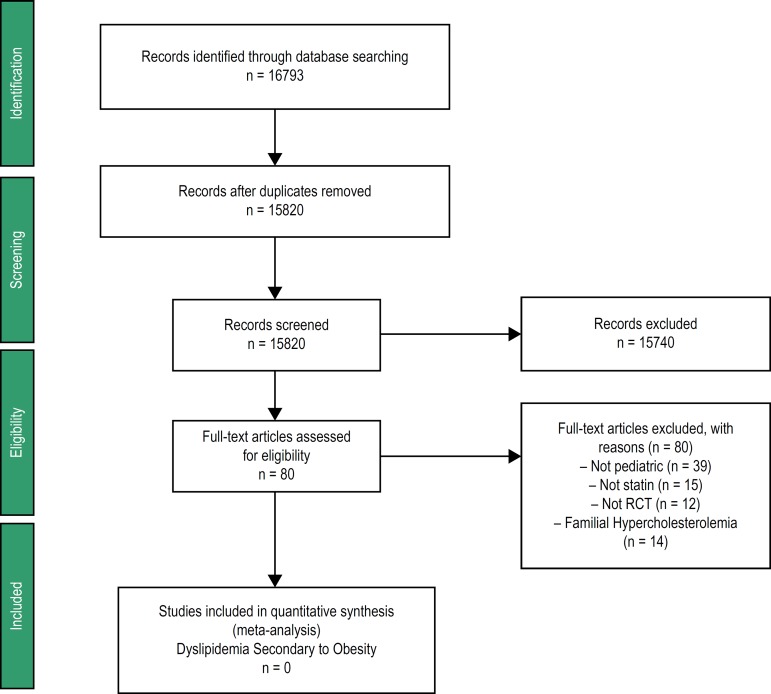
Flow diagram of the studies included. RCT: randomized clinical trial.

We identified 16793 citations from the electronic search of the databases from inception to February 2016. After duplicate studies were removed, 15820 studies were subjected to title and abstract screening. We excluded 15740 studies and 80 studies were subjected to full-text review. We did not include any randomized clical trial about DSO in children, and all 80 articles of full-texts were excluded for the reasons: 39 studies on non-pediatric population (subjects aged 18 to 80 years), 15 studies did not use treatment with statins, 12 studies did not have the design of a randomized clinical trial, and 14 randomized clinical trials evaluated children and adolescents with familial hypercholesterolemia (FH), involving a total of 2347 individuals (Figure 1).

## Discussion

Dyslipidemia secondary to obesity in children and adolescents is increasingly prevalent in clinical practice. However, the present review did not retrieve specific evidence about drug therapy in this group.

In this type of dyslipidemia, the most common lipid alterations are low HDL-C and elevated triglycerides (TG) secondary to insulin resistance syndrome.^7^ High TC levels may be associated with these conditions, but cannot be considered the most important factor. Steinberg et al.^8^ showed that the degree of insulin resistance explains a significant proportion of variation in the levels of TG, LDL-C, and HDL-C, and Stan et al.^9^ estimated a 10% prevalence of small dense LDL (sdLDL) particles in children showing insulin resistance compared to 1% in those without insulin resistance. As recommended by the Expert Panel,^5^ low saturated-fat and cholesterol diet is the first approach to lower TC and LDL-C levels, to reduce obesity and insulin resistance, and to prevent the development of atherosclerosis. These recommendations^8^ confirm that primary prevention in children with dyslipidemia involves lifestyle modification. In childhood, the construction of healthy eating habits must be emphasized, since early preference patterns have a long-term influence on dietary intake later in life.^10,11^ To provide information about nutrition is, therefore, an important part of the routine visits.^12,13^ However, neither assessment of the patient’s nutritional status nor discussion of dietary habits seem to be performed systematically.^14^ Physicians often point to the lack of knowledge in this area as one of the main limitations to this practice.^15-17^

On the other hand, obese children may also suffer from FH, that is phenotypically diagnosed by the presence of high levels of LDL-C and a family history of premature cardiovascular disease and/or high cholesterol at baseline in one of the parents and/or a mutation that causes FH.^18-20^ After dietary intervention, any child with LDL-C ≥ 5 mmol/L (190 mg/dL) has high probability of having genetic FH. A family history of premature cardiovascular disease in close relatives and/or high cholesterol levels at baseline in a parent, LDL-C ≥ 4 mmol/L (160 mg/dL) are also indicative of a high probability of genetic FH. The detection of a pathogenic mutation, usually in the LDLR gene, is the gold-standard diagnosis test for FH. The LDL-C levels must be measured at least twice in 3 months to confirm the diagnosis of FH.^21^

The maintenance of a healthy lifestyle and statin treatment (age 8 to 10 years) are proposed as the main interventions to control heterozygous FH (HeFH). The target of LDL-C for children is 3.5 mmol/L (130 mg/dL) if > 10 years old, or, ideally, to reduce 50% of the baseline level among children aged 8 to 10 years, especially with an extremely high LDL-C, high levels of lipoprotein(a), family history of premature cardiovascular disease or others cardiovascular risk factors, balanced against the risk of long-term adverse effect of treatment.^18,21-23^ Statins have shown better effects on major cardiovascular outcomes, justifying their use despite their still unknown side effects when used for more than 2 years in children.^24^

The inhibitors of HMG-CoA reductase have shown repeatedly in random controlled studies to effectively reduce coronary morbidity and mortality in adults at high risk. As a result, statins have become one of the most prescribed drugs for adults in the world.^25^ In adults, statins have proved to effectively reduce both LDL-C levels and vascular events.^26,27^

At usual doses, statins are a remarkably safe drug group. Few reports exist about serious adverse gastrointestinal events, and hepatic transaminases and creatine-phosphokinase elevation. However, evidence for their use in children still lacks.^28^ Children with serious lipid abnormalities due to genetic disorders may meet the criteria for drug therapy with the statins commonly used in adults. In the last few years, reports about the short-term safety of some of these drugs in this group have been published.^29-31^

All statins recommended by the US Food and Drug Administration (FDA)^23^ have been approved for children with FH and some other primary or genetic dyslipidemia causes. Data about cholesterol reduction in other groups of children were insufficient.^32^ The statins used to treat children with HeFH are approved by the FDA or used in treatments based on cholesterol-lowering studies in children with HeFH.^33^ For other dyslipidemia causes in children, the focus should be on the diet and treating subjacent metabolic disorders.^34^ Treatment may be started earlier in severe cases.^35^

Effectiveness and safety are similar in both children with genetic disorders and children with DSO in the short term. However, concerns about long-term safety still remain.^36,37^ None of these studies cited above had a long-term follow up, and none of them described potential late collateral effects of early therapy for cholesterol reduction or delay in cardiovascular outcomes.^38^ Kusters et al.^39^ have reported the longest follow-up in children with FH treated with statins. Long-term treatment with statins started during childhood in patients with FH was associated with the normalization of the ntima-media thickness progression. No serious adverse event was reported during the 10-year follow-up. Braamskamp et al.^40^ have published the first study that evaluated the long-term effect of statin treatment started in childhood on the plasma of gonadal steroid hormones, gonadotropins and dehydroepiandrosterone in young adults with FH. After 10 years of statin treatment, the concentrations of testosterone, estradiol, luteinizing hormone and follicle stimulating hormone in those patients with FH were within the reference range when compared with non-affected siblings.

Before starting the widespread use of statins in children with secondary dyslipidemias, ideally studies should establish that statins can reduce total morbidity and mortality in the long-term. There must also be a logical progression of studies addressing primary prevention, from the oldest to the youngest. The use of statins for primary prevention in adults with secondary hyperlipidemia is currently under debate. The introduction of statins at an earlier age may offer the possibility of greater risk reduction than the one currently observed in studies with adults, but to this date this hypothesis remains highly speculative.

## Conclusions

In our search, we found no randomized clinical trial addressing the use of statin therapy in children and adolescents with DSO. All studies retrieved had been performed in patients with FH.

The usual practice of extrapolating findings from studies in genetic dyslipidemia ignores the differences in long-term cardiovascular risks and long-term drug treatment risks, when compared to recommendation of lifestyle changes. Randomized clinical trials are needed to understand drug treatment in secondary dyslipidemia.
